# Prebiotics Mediate Microbial Interactions in a Consortium of the Infant Gut Microbiome

**DOI:** 10.3390/ijms18102095

**Published:** 2017-10-04

**Authors:** Daniel A. Medina, Francisco Pinto, Aline Ovalle, Pamela Thomson, Daniel Garrido

**Affiliations:** Department of Chemical and Bioprocess Engineering, School of Engineering, Pontificia Universidad Católica de Chile, Av. Vicuña Mackenna 4860, Santiago 7820436, Chile; damedinas@uc.cl (D.A.M.); fmpinto@uc.cl (F.P.); amovalle@uc.cl (A.O.); ptm_thomson@hotmail.com (P.T.)

**Keywords:** prebiotics, human milk oligosaccharides, intestinal microbiome, microbial interactions

## Abstract

Composition of the gut microbiome is influenced by diet. Milk or formula oligosaccharides act as prebiotics, bioactives that promote the growth of beneficial gut microbes. The influence of prebiotics on microbial interactions is not well understood. Here we investigated the transformation of prebiotics by a consortium of four representative species of the infant gut microbiome, and how their interactions changed with dietary substrates. First, we optimized a culture medium resembling certain infant gut parameters. A consortium containing *Bifidobacterium longum* subsp. *infantis*, *Bacteroides vulgatus*, *Escherichia coli* and *Lactobacillus acidophilus* was grown on fructooligosaccharides (FOS) or 2′-fucosyllactose (2FL) in mono- or co-culture. While *Bi. infantis* and *Ba. vulgatus* dominated growth on 2FL, their combined growth was reduced. Besides, interaction coefficients indicated strong competition, especially on FOS. While FOS was rapidly consumed by the consortium, *B. infantis* was the only microbe displaying significant consumption of 2FL. Acid production by the consortium resembled the metabolism of microorganisms dominating growth in each substrate. Finally, the consortium was tested in a bioreactor, observing similar predominance but more pronounced acid production and substrate consumption. This study indicates that the chemical nature of prebiotics modulate microbial interactions in a consortium of infant gut species.

## 1. Introduction

The intestinal microbiome is a complex and large community of microorganisms that reaches one of the highest cell densities recorded [1]. Not surprisingly the gut microbiome has a great impact on host health, especially regarding our metabolism and immune system [2]. The establishment and composition of the gut microbiome has been shown to be critical later in life, with certain diseases such as asthma, allergies, diabetes and obesity being linked to microbiome dysbiosis [3,4].

Diet is a key player in shaping the early gut microbiome. Breast milk is the gold standard in infant nutrition, and breastfeeding confers the infant several health benefits [5]. One of these benefits is the assembly of a healthy gut microbiome [6], which has been attributed to human milk oligosaccharides (HMO), present in large quantities in breast milk [7]. HMO are complex carbohydrates that transit the gastrointestinal tract and, in addition to other roles, act as prebiotics, selectively stimulating the growth of beneficial gut bacteria [8,9]. Certain HMO, such as lacto-*N*-tetraose and 2′-fucosyllactose (2FL; Fucα2-1Galβ1-4Glc), are remarkably abundant in breast milk [10].

Not all infants are breast-fed and infant formula, usually produced from bovine milk, is used as a substitute [5]. Prebiotics are common food ingredients added to infant formula. Fructooligosaccharides (FOS) are a mixture of linear polymers of fructose in β2-1 linkage with a terminal glucose and a degree of polymerization (DP) of 3 to 6 [11]. FOS, together with inulin which has a higher DP, are obtained from certain plant roots such as chicory. Despite their wide use in foods, these prebiotics do not fully match the structural complexity of HMO.

Breast-fed infants possess a distinct microbiome characterized by a low diversity and a dominance of *Bifidobacterium* species [12,13]. Bifidobacteria are common members of the gut microbiome especially in infants [14]. Their presence is normally regarded as beneficial due to their increased acid production. Species such as *Bi. breve*, *Bi. bifidum* and *Bi. longum* subsp. *infantis* (*Bi. infantis*) are commonly found in this environment [15,16]. These species possess the ability to utilize HMO, and the molecular mechanisms involved have been described for a few species [17,18,19]. The dominance of bifidobacteria is usually characterized by higher amounts of acetate in feces compared to formula-fed infants [8]. Acetate is associated with several health benefits including pathogen deflection in the gut [20].

The formula-fed gut microbiome is characterized by a higher microbial diversity [21], with higher abundances of *C. difficile*, *B. adolescentis* and certain species of *Proteobacteria* [22]. Less attention has been paid to the relevance of other species in the infant gut microbiome, such as those from phylum *Bacteroidetes* (*Bacteroides thetaiotaomicron*, *Bacteroides vulgatus*), *Firmicutes* (*Lactobacillus* spp., *Clostridium* spp.) and *Proteobacteria* (*Escherichia coli*, *Enterococcus* spp.) [8,21,23,24,25]. In particular, *Bacteroides* spp. have a wide preference for complex polysaccharides, acting as primary fermenters in the microbiome [26]. Their glycolytic machinery is based on polysaccharide utilization loci, and several *Bacteroides* have been shown to utilize HMO, FOS, mucin and multiple plant-derived oligo and polysaccharides [27,28,29]. *Lactobacillus* species are less abundant in the infant gut microbiome, however they are still a focus of enrichment by prebiotics such as FOS and inulin [30]. These species display a metabolism that targets simple sugars. Finally, *E. coli* and other enterobacteria are commensal microbes that produce certain vitamins for the host and their metabolism is also adapted to the utilization of simple carbohydrates [31].

Considering the importance of the early colonization of the gut microbiome in health, microbial interactions are essential for microbiome assembly. Dominant ecological interactions in the microbiome are competition and cooperation [32]. Interactions among gut bacteria could be exemplified by competitive exclusion during colonization [33], biofilm formation [34] or quorum sensing [35]. Cross-feeding appears to be common in gut species, where bacteria release breakdown products, allowing other species to grow on simpler oligosaccharides [36]. These interactions could also involve cross-feeding of products of metabolism such as lactate and acetate (short chain fatty acids or SCFA) [37,38]. All these metabolic interactions are probably dependent on the chemical structure of dietary glycans reaching the infant gut.

In this work, a consortium of four representative bacteria of the infant gut microbiome was used to study the effect of two prebiotics on microbial interactions in the microbiome. For this, we first optimized a semi-defined culture medium that allowed good growth of gut microbes, simulating certain conditions prevalent in the infant gut. Later, the impact of FOS or 2FL on interactions within the consortium was evaluated, determining the abundance of each member, their substrate consumption and the production of major fermentation products. Finally, the consortium was evaluated in a pH/oxygen controlled stirred bioreactor during growth on these prebiotics.

## 2. Results

### 2.1. mZMB Optimization

In order to evaluate the interactions among representative infant gut microbes, we first designed a culture medium that allows good simultaneous growth of representative gut bacteria, including some physical and chemical parameters of the infant gut. We started with ZMB-1 [39], a chemically defined medium that was originally optimized for the growth of lactic acid bacteria. The modified medium contains bile salts and mucin, adjusting pH to 5.5 [40]. Instead of single amino acids as a nitrogen source, we chose to include tryptone and peptone, enzymatic digests from bovine casein, that resemble partially degraded proteins as found in the large intestine [41]. Initial concentrations of peptone and tryptone, equivalent to the total amino acid content in ZMB-1, were estimated to be 2.5 g·L^−1^ and 8.1 g·L^−1^ respectively.

**Figure 1:**
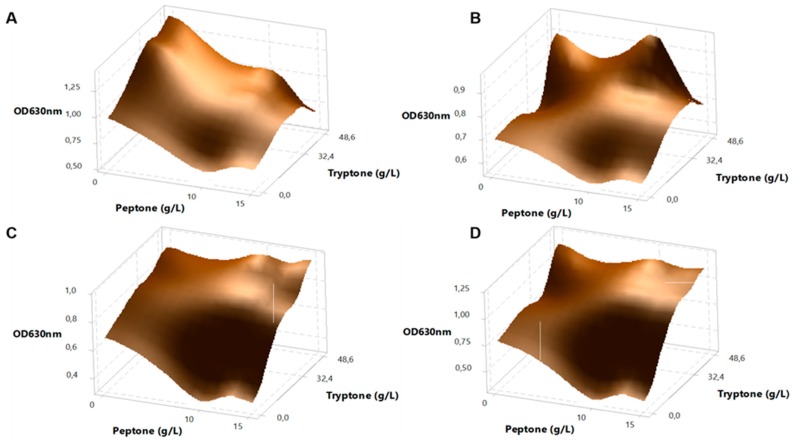
Surface plots of the optimization of protein concentrations in mZMB media. For the four bacteria in the consortium, different tryptone and peptone concentrations were assayed, and maximum optical density (OD_630_) values were obtained. (**A**) *Bi. infantis*; (**B**) *Ba. vulgatus*; (**C**) *E. coli*; (**D**) *L. acidophilus*.

Later, an experimental design varying these concentrations was used to maximize growth of gut bacteria in this media. This initial design had two variables (tryptone and peptone), and was performed in three levels: absence, four and six times each variable using glucose as the carbon source. Figure 1 shows surface plots for *Bi. infantis*, *Ba. vulgatus*, *E. coli* and *L. acidophilus*. Tryptone had a greater impact increasing cell growth in all four cases, even in the absence of peptone. Moreover, when using six times the starting concentration of tryptone, additional peptone appeared to have a negative impact on growth in all cases. We then chose the lowest concentration of tryptone that allowed the highest growth (Figure 1) and left peptone out of this medium (Table S1).

### 2.2. Abundance in Co-Culture Experiments

The same consortium of species mentioned above ((*Bi. infantis* (Bi); *Ba. vulgatus* (Bv); *E. coli* (Ec) and *L. acidophilus* (La)) was studied for interactions in two prebiotics. Briefly, bacteria were cultured in mono or paired cultures in mZMB, supplemented with 1% of either 2FL or FOS (Figure S1). Experiments were run for 48 h and samples were obtained every 12 h. An experiment containing all four bacteria on each carbon source was also included. Microbial abundance was determined by qPCR (Figures S2 and S3).

**Figure 2:**
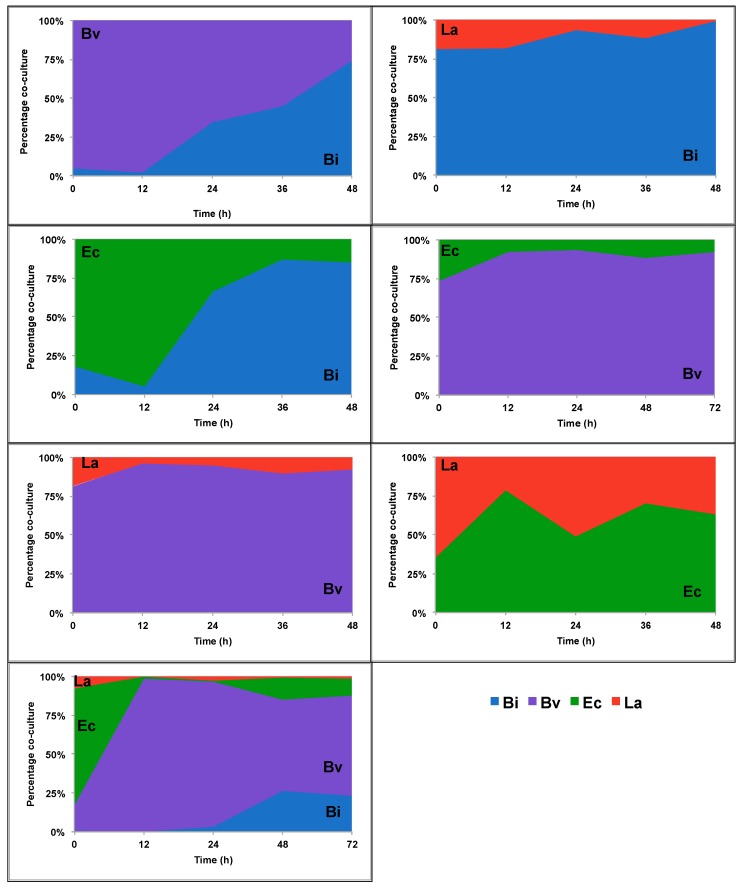
Bacterial abundance in co-culture experiments using 2′-fucosyllactose (2FL). Co-cultures of Bi (*Bi. infantis*), Ba (*Ba. vulgatus*), Ec (*E. coli*) and La (*L. acidophilus*) were grown as shown in the legend. Cell copy numbers were determined every 12 h. Percentages represent the proportion of the cell copies for each bacterium in each combination.

**Figure 3:**
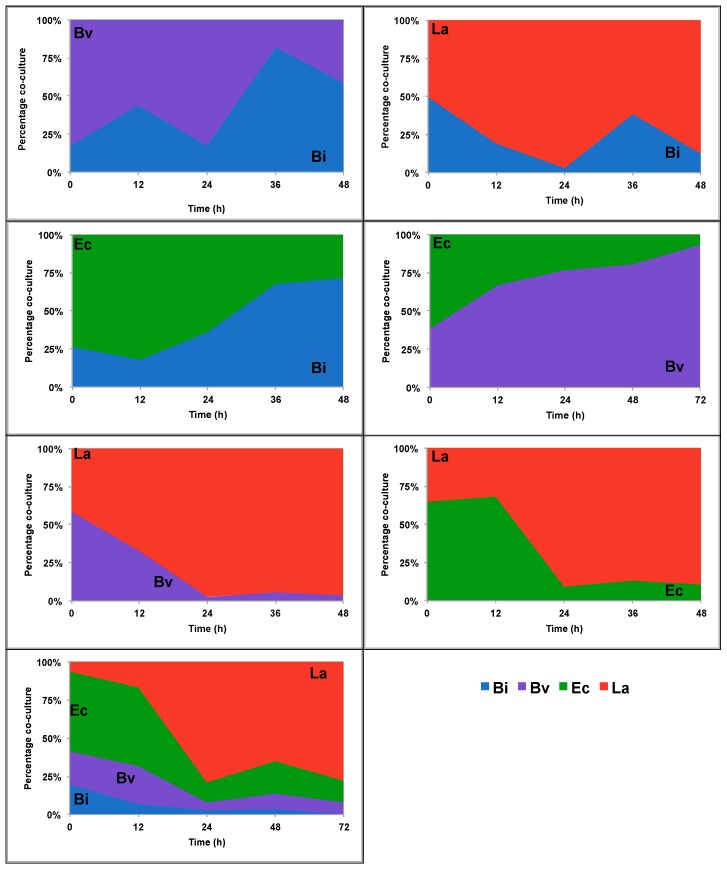
Bacterial abundance in co-culture experiments using fructooligosaccharides (FOS). Co-cultures of Bi (*Bi. infantis*), Ba (*Ba. vulgatus*), Ec (*E. coli*) and La (*L. acidophilus*) were grown as shown in the legend. Cell copy numbers were determined every 12 h. Percentages represent the proportion of the cell copies for each bacterium in each combination.

To determine the actual contribution of a species in a co-culture, we added cell counts of both members in a combination (100%) and determined the relative proportion of each bacterium (Figure 2 and Figure 3). On 2FL, *Bi. infantis* and *Ba. vulgatus* appeared to dominate the fermentations over *E. coli* and *L. acidophilus* (Figure 2). While *Bi. infantis* appeared to outcompete *Ba. vulgatus* at the end of their co-culture, growth of the whole consortium was dominated by *Ba. vulgatus* followed by *Bi. infantis*.

Interestingly, *L. acidophilus* largely dominated all combinations during combined growth on FOS. This is remarkable since *Bi. infantis* and *Ba. vulgatus* are also capable of growing using FOS as the sole carbon source (Figure S2). After *L. acidophilus*, *Bi. infantis* outcompeted *Ba. vulgatus* and *E. coli* (Figure 3).

**Figure 4:**
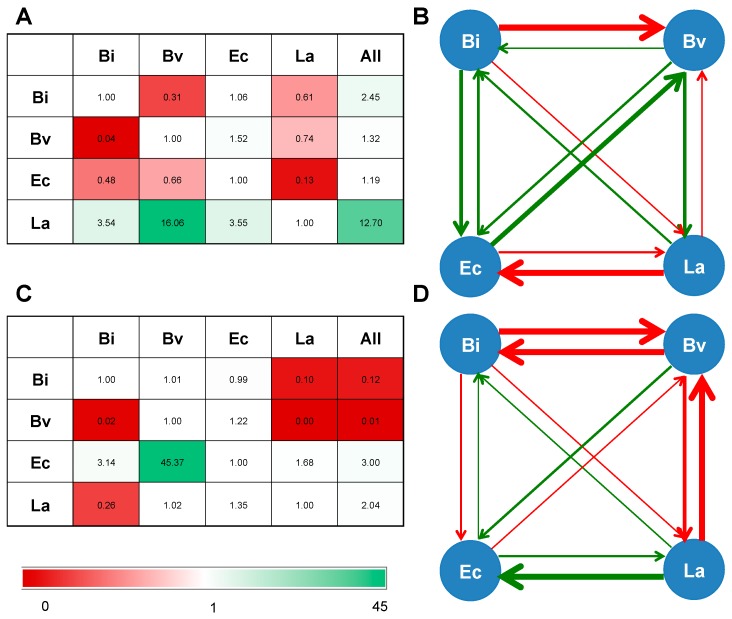
Interactions among members of the consortium. (**A**,**C**) Effect of microbes in the first row on the maximum growth of microbes in the first column. This was calculated for data on growth on 2FL (**A**) or FOS (**C**). A value of 1 means no effect, and legend indicate this normalized ratio; (**B**,**D**) effect of bacteria in the consortium on the growth rate of one microbe, in 2FL (**B**) or FOS (**D**). Arrows indicate the direction of this effect. Red indicates a negative effect on growth rate (competition), and green indicate a positive value (cooperation). Width of arrows are proportional to the calculated effect (shown in Table S4).

To evaluate the impact of the presence of one bacterium on another’s growth, we determined two parameters. First, we calculated the ratio of the maximum cell copy number of one species in co-culture over the maximum cell number of the same species in monoculture (Figure 4A,C). In addition, we obtained the ratio of growth rate of any bacterium in co-culture, to the growth rate of the same bacterium in mono-culture (Table S3). The effect of one bacterium on another’s growth rate is proportional to the interaction coefficients of the Lotka-Volterra equation [42], and these values (Table S3) are pictured in Figure 4B,D.

Maximum growth of both *Bi. infantis* and *Ba. vulgatus* was reduced several fold in their combination on 2FL (Figure 4A). Additionally, growth rate of *Ba. vulgatus* appeared slower in presence of *Bi. infantis* (Figure 4B). Similarly, *Bi. infantis* and *L. acidophilus* reduced the growth of *E. coli* on 2FL. Finally, *L. acidophilus* reduced several fold *E. coli* maximum growth and its growth rate in 2FL (Figure 4A). In general, all bacteria showed higher growth rates in presence of the complete consortium (Table S3).

Interactions on FOS appeared to be stronger and more negative (Figure 4C,D). Maximum growth of *Bi. infantis* and *Ba. vulgatus* was smaller in their co-culture on this substrate. Moreover, growth of *Bi. infantis* and *Ba. vulgatus* in FOS appeared to be reduced several fold in the presence of *L. acidophilus* or the whole consortium (Figure 4D). This was also shown by a reduction in growth rate in these co-cultures. Two important positive interactions were observed in FOS co-cultures: *E. coli* displayed an increased growth in the presence of *Ba. vulgatus*, and *L. acidophilus* appeared to increase *E. coli* growth rate. These results suggest that *E. coli* benefits from the activities of the other microbes, without altering the growth of other bacteria (Figure 4).

### 2.3. Effect of Co-Culture on Prebiotic Consumption

**Figure 5:**
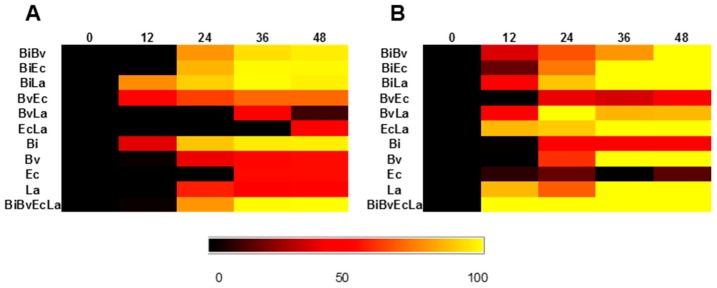
Consumption of 2FL (**A**) or FOS (**B**) in mono or co-culture. Values were calculated using the phenol-sulphuric method and expressed as percentage consumption relative to initial concentration. Bi: *Bi. infantis*; Bv: *Ba. vulgatus*; Ec: *E. coli*; La: *L. acidophilus*.

To determine if the observed growth and interactions observed are related to substrate consumption, total carbohydrate consumption was determined (Figure 5). As expected from abundance data, *Bi. infantis* in monoculture or its co-cultures displayed full consumption of 2FL. Co-culture of the four species on 2FL also displayed a fast consumption (Figure 5A). Interestingly, *Ba. vulgatus* in monoculture or its combinations displayed a poor consumption of 2FL, less than 50%. When evaluating protein concentration, we found that co-cultures of *Ba. vulgatus* and *E. coli* display an important depletion of proteins (Table S4), suggesting that their increase in cell counts is in part due to protein fermentation.

Consumption of FOS was more pronounced than 2FL, with several combinations of *L. acidophilus* and *Bi. infantis* reaching full consumption at 12–24 h (Figure 5B). Protein utilization was not majorly observed during FOS consumption (Table S4).

### 2.4. Production of Short Chain Fatty Acids (SCFA) in Co-Cultures

**Figure 6:**
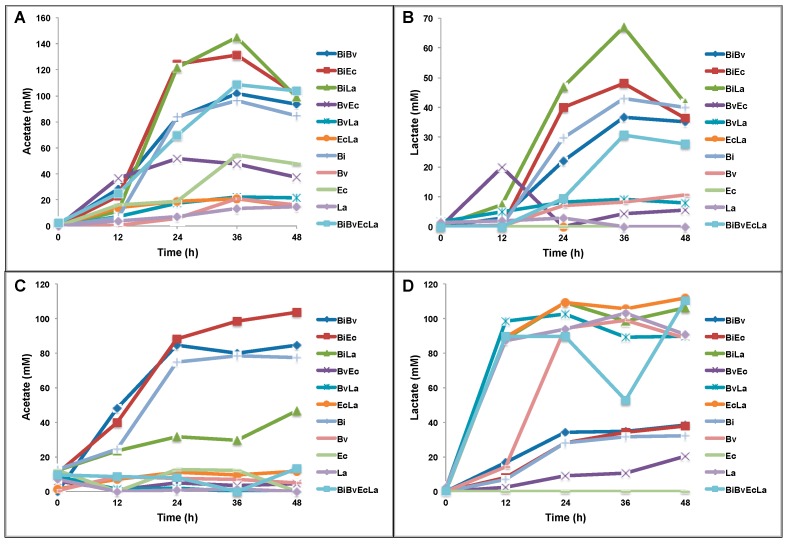
Acetate and lactate production during growth in 2FL (**A**,**B**) or FOS (**C**,**D**). Data was obtained for mono and co-cultures of Bi (*Bi. infantis*), Bv (*Ba. vulgatus*), Ec (*E. coli*) and La (*L. acidophilus*). The acids were quantified from bacterial supernatants every 12 h of incubation and expressed in mM.

We later quantified the production of acetate and lactate, major fermentation products in the consortium, after 48 h in mono and co-culture in both prebiotics (Figure 6). In general, acid production was higher during growth on FOS than 2FL, and co-cultures of the four bacteria did not necessarily produce more acid than mono or co-cultures (Figure 6).

On 2FL, mono and co-cultures of *Bi. infantis* produced more acetate and lactate, in concordance with the ability *Bi. infantis* of fermenting this HMO (Figure 6A,B). Interestingly, the complete consortium produced less acetate or lactate compared to combinations BiEc or BiLa. While *Ba. vulgatus* appeared to dominate 2FL growth (Figure 2), its production of acetate or lactate was scarce and it is possible that other fermentation products are being produced by this bacterium (Figure 6A,B).

Acetate production during growth on FOS was more pronounced in cultures BiEc, BiBv and Bi (Figure 6C). Interestingly, acetate was less abundant in *L. acidophilus* combinations including BiLa, which is consistent with a higher abundance of *L. acidophilus* compared to *Bi. infantis*. In order with this observation, lactate was produced at high concentrations in *L. acidophilus* mono or co-cultures (Figure 6D). Single growth of *Ba. vulgatus* on FOS also resulted in high concentrations of lactate, much more compared to growth on 2FL (Figure 6D). Moreover, this increased production of lactate by *Ba. vulgatus* was not observed in the presence of *E. coli* or *Bi. infantis*.

### 2.5. Validation in Bioreactor

**Figure 7:**
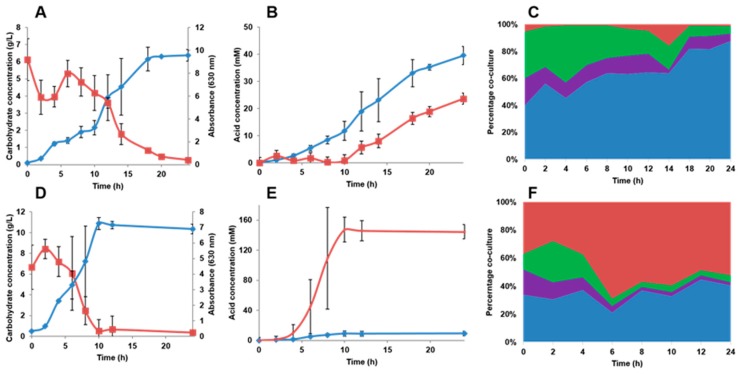
Batch cultures of the consortium in 2FL or FOS. Results represent the average of two 24 h fermentations. (**A**,**D**) Total biomass production (blue) and substrate consumption (red) during growth in 2FL or FOS respectively. Error bars indicate the standard error mean; (**B**,**E**) acetate (blue) and lactate (red) production in the fermentations using either 2FL or FOS; (**C**,**F**) relative abundance of each member of the consortium during growth in 2FL (**C**) or FOS (**F**). Red: *L. acidophilus*; green: *E. coli*; purple: *Ba. vulgatus*; blue: *Bi. infantis*.

The four-species consortium was finally cultured in an anaerobic bioreactor. This set-up more optimal culture conditions, including agitation and fixed pH at 5.5. In this bioreactor, biomass values reached an OD_630_ over 7 (2FL) or 10 (FOS) in 24 h (Figure 7). Both substrates were completely consumed at the end of the fermentations. Interestingly acetate was produced before lactate by the consortium (Figure 7B). However, the amount of acetate and lactate was lower compared to the microplate assay, probably due to the fixed pH used in the bioreactor. Acetate and lactate production correlates with the predominance of *Bi. infantis* in the consortium, which reached up to 90% of total gene counts in the consortium in the bioreactor (Figure 7C).

Growth of the consortium in FOS led to an increased production of lactate, which is in order with a predominance of *L. acidophilus* under these conditions (Figure 7E,F). Similar to the above assays, *Ba. vulgatus* does not seem competitive in FOS utilization in the presence of other species such as *Bi. infantis* and *L. acidophilus*.

## 3. Discussion

Several studies regarding the utilization of prebiotics by gut microbes have been performed using single strains or fecal samples using batch systems [37,43,44,45,46]. However, it is likely that gut microbes deploy complex mechanisms for targeting dietary substrates, which could include competition or cooperation. Therefore, microbial utilization of prebiotics is probably dependent on microbial interactions. In this study we used a simplified consortium of infant gut microbes [24], to evaluate the effect of two oligosaccharides on the microbial interactions of this consortium.

To better address interactions in this environment, it is important to recapitulate the conditions prevalent in the infant gut. The culture medium mZMB favored the growth of gut bacteria in a more acidic environment and in the presence of mucin and bile salts, important modulators of the gut microbiome. The optimal medium concentration for the microorganisms tested contained tryptone, a digest from milk casein, which provides partially degraded peptides. This is important to consider since the luminal colon does not contain single amino acids [41].

The behavior of the consortium in the presence of 2FL or FOS could be comparable to in vivo studies, where milk oligosaccharides support a predominance of bifidobacteria, while formula feeding results in a more diverse microbiome [8,23]. 2FL is one of the most abundant HMO in breast milk, and it has been shown to modulate microbiome composition and fecal metabolic profiles [8,18]. *Bi. infantis* dominated growth on 2FL, and its combinations completely utilized this substrate and produced high amounts of acetate and lactate, which is expected from *Bi. infantis* metabolic activity. We observed that *Bi. infantis* also limited *Ba. vulgatus* growth in their combined culture, decreasing its maximum growth and growth rate. In the presence of *Bi. infantis*, *Ba. vulgatus* appeared to minimally consume 2FL. The growth of this strain in vitro in HMO has been shown not to be vigorous [47], and while its genome sequence contains a fucosidase gene, it appears to lack certain fucose metabolism enzymes [48]. *Ba. vulgatus* also caused an important decrease in tryptone in the media. In order with this, *Ba. vulgatus* produced small amounts of acetate and lactate, suggesting that its metabolism could be releasing other products into the media.

The interactions within the consortium were more pronounced during growth on FOS. This is probably a reflection of the ability of all members to consume the substrate or its breakdown products. Fructose utilization is common among gut bacteria, and several bacteria can feed on FOS [30]. In our system, all species except *E. coli* were able to consume this substrate. *L. acidophilus* dominated FOS utilization [49], limiting several-fold the growth of *Bi. infantis* and *Ba. vulgatus*, which in monoculture consumed the substrate. Growth rate in monoculture could be a good predictor of predominance in our system, since bacteria with higher growth rates on a substrate predominate over other microbes with lower growth rates (Table S3, Figure 2 and Figure 3). However, there are several interactions not related to this observation. For example, *Ba. vulgatus* stimulates growth of *L. acidophilus* several-fold during 2FL utilization, and *Ba. vulgatus* also stimulates growth of *E. coli* during FOS consumption (Figure 4).

It is likely that some of these interactions are mediated by cross-feeding. Mechanistically, certain *Bacteroides* species release simple monosaccharides to the lumen after polysaccharide breakdown [50,51]. In turn, these carbohydrates could be utilized by other microbes [52]. In our case, we hypothesized that *Ba. vulgatus* could release lactose and fucose to the media, or fructose, during growth on 2FL or FOS respectively. In addition, in some cases acetate and lactate produced by *Bi. infantis* and *L. acidophilus* could be used by *Ba. vulgatus* or *E. coli*. This could explain in part the higher *Ba. vulgatus* and *E. coli* cell counts in 2FL without being strong 2FL consumers.

An important interaction was observed during FOS consumption, where *Ba. vulgatus* and *Bi. infantis. infantis* displayed a strong competition. Certain co-culture studies indicate a negative effect of *Bi. longum* on *Ba. thetaiotaomicron*, with the latter expanding its metabolic capabilities to utilize other substrates [53]. Some *Bacteroides* can release antimicrobial proteins against other members in this genus [54], and *Ba. ovatus* enhances the growth of *Ba. vulgatus* through metabolic sharing [55]. A suppressive effect from *Bi. infantis* has been previously reported on *Ba. vulgatus* [56]. In this study, their interaction altered their FOS consumption profiles, which were more reduced for *Bi. infantis* in co-culture, and also limited the amount of acetate produced in co-culture BiBv. Which molecules or mechanisms are mediating these interactions is not clear. An additional factor probably limiting *Ba. vulgatus* growth is pH, considering that in general *Bacteroides* species are more sensitive to pH drops [57].

## 4. Materials and Methods

### 4.1. Bacteria and Media

Strains used in this study were obtained from the UC Davis Viticulture & Enology Culture Collection (*Bifidobacterium longum subsp. infantis* ATCC 15697, *Lactobacillus acidophilus* ATCC 4356, *Escherichia coli* K12), and the American Type Culture Collection (*Bacteroides vulgatus* ATCC 8482; Manassas, VA, USA). For routine experiments, bifidobacteria were grown on de Mann-Rogose-Sharp (MRS) broth supplemented with 0.05% *w*/*v* l-cysteine (Loba Chemie, Maharashtra, India) and incubated for 48 h at 37 °C in an anaerobic jar (Anaerocult, Merck, Darmstadt, Germany) with anaerobic packs (Gaspak EM, Becton-Dickinson, Franklin Lakes, NJ, USA). Lactobacilli were grown under the same conditions but without l-cysteine. *Bacteroides* strains were grown anaerobically using Reinforced *Clostridium* Medium (Becton-Dickinson) supplemented with 1 g·L^−1^ l-cysteine. All media were pre-reduced in an anaerobic jar overnight before inoculation. *E. coli* was routinely grown on Standards Methods Broth (Becton-Dickinson). Prior to each assay all bacteria were subcultured twice. All chemicals were acquired from Merck, Sigma or Calbiochem.

### 4.2. Adaptation and Optimization of mZMB

We started from a chemically defined media, ZMB-1 [39], which has been used to culture lactic acid bacteria and gut microbes [47,58]. This medium contains 22 major groups. From the original composition, acetate potassium, Tween 80 and MOPS were not included, and pH was adjusted to 5.5. Groups containing single amino acids were excluded, and replaced by an equivalent amount of tryptone and peptone (Becton-Dickinson). This amount was estimated based on the amino acid composition of tryptone and peptone (Becton-Dickinson), and by minimizing the lineal combination of these components to match ZMB-1 amino acid composition, using the tool Solver in Excel and Equation (1).

(1) $$\underset{a,b}{\min}\;(a\times \vec{P}+b\times \vec{T})\;\geq \vec{ZMB1}\;\;$$

To optimize protein content of the final medium to allow high bacterial growth we sought to find the minimum values of the variables of tryptone and peptone (*a* = 2.5 and *b* = 8.1 g·L^−1^ respectively), and experiments were prepared in their absence, or with 4 or 6 times the initial concentrations. These experiments contained 2% glucose as the carbon source. Fixed groups and glucose were filtered separately, and tryptone and peptone were autoclaved and mixed with the other components under sterile conditions. Finally, culture medium with varying amounts of protein sources were aliquoted in 96-well plates, and inoculated with 2% of a fresh culture of either *Bi. infantis*, *Ba. vulgatus*, *E. coli* or *L. acidophilus*. To prevent evaporation, wells were covered with sterile mineral oil. Plates were incubated anaerobically at 37 °C for 72 h in anaerobic jars, and final optical density (OD) at 630 nm was determined in a microplate reader (Tecan Trading AG, Mod. Infinite M200 PRO, Männedorf, Switzerland). Maximum OD values were used in a surface response analysis using Minitab 17 Statistical Software.

Negative controls with no carbon source and without bacteria were included, as well as a positive control using rich growth medium for every microorganism as described above. Bacteria were inoculated as described and incubations were carried out at 37 °C in a microplate reader inside an anaerobic chamber with periodic injection of pure nitrogen. Oxygen levels were kept under 0.1% in the chamber and cell growth was monitored every 30 min by reading optical density at 630 nm. Each measurement was preceded by 10 s shaking at maximum speed. Two biological replicates and three technical replicates were included for each species.

### 4.3. Co-Culture Experiments

The optimized formulation of mZMB (Table S1) was supplemented with either 1% FOS (Raftilose Synergy 1, Orafti, Malvern, PA, USA) or 1% 2FL (Jennewein Biotechnologie, Rheinbreitbach, Germany). FOS used displayed a chain length of 3–7, as observed in thin layer chromatography plates. The following bacterial combinations were inoculated: single cultures of *Bi. infantis* (Bi), *Ba. vulgatus* (Bv), *E. coli* (Ec) and *L. acidophilus* (La); and co-cultures BiBv, BiEc, BiLa, BvEc, BvLa and EcLa. An experiment with all four bacteria (BiBvEcLa) and a negative control with no bacteria were also included. Overnight cultures of each microorganism were washed in sterile and reduced mZMB, and diluted in the same medium in order to obtain a similar OD for each bacteria at the start of the experiment. 1 mL of each diluted culture was used to inoculate 10 mL of mZMB containing either FOS or 2FL. This experiment was performed in duplicate. Volumes of 200 μL of inoculated mZMB were aliquoted in 96 well sterile microplates, covered with 30 μL of sterile mineral oil, and incubated anaerobically at 37 °C for either 12, 24, 36 or 48 h. Samples were recovered from the microplates at those times and centrifuged at 12,000× *g* for 2 min. Pellets and supernatants were stored at −20 °C until use.

### 4.4. Batch Bioreactor Co-Cultures

A 250 mL bioreactor (Mini-bio Applikon Biotechnology, JG Delft, The Netherlands) was used for culturing the four-species consortium. The bioreactor was set to 100 rpm agitation, 37 °C and pH was set at 5.5 with automatic injection of 3N HCl or 3N NaOH. Pure N_2_ was automatically injected when O_2_ concentration measured was above 1 ppm. Culture medium used was mZMB, prepared as described above, and supplemented with either 1% FOS or 1% 2FL. Each substrate was tested in two independent replicates. Under sterile conditions, every microorganism was inoculated to an initial OD_630_ of 0.05. Foam was controlled using polydimethylsiloxane base (Winkler, Santiago, Chile). 2 mL from the bioreactor were sampled every 2 h (up to 24 h) and centrifuged at 4000× *g* for 5 min. Supernatants were stored at −20 °C for carbohydrate and acid analysis. Pellets were stored for DNA extraction, which was later quantified and diluted to 10 ng/μL for qPCR assays as described above (AriaMx Realtime PCR System, Agilent Technologies, Santa Clara, CA, USA).

### 4.5. DNA Extraction

Total DNA was extracted from cell pellets using a modified version of a phenol chloroform isoamyl protocol [59]. The protocol is optimized to isolate DNA from Gram+ and Gram− bacteria. Briefly, pellets were resuspended in NaCl–TRIS–EDTA buffer (200mM NaCl, 200 mM TRIS and 20 mM EDTA) with 0.4 mg of lysozyme (Amresco, Toronto, Canada). Resuspended cells were incubated for 30 min at 37 °C. The suspension was transferred into tubes with 0.4 mL of phenol:chloroform:isoamilic-alcohol 25:24:1 (pH 8) and sodium dodecyl sulphate 3% in addition to 0.1 μL of sterile acid-washed glass beads (Sigma, St. Louis, MO, USA). Cells were disrupted for 5 min using a Disruptor Genie (Scientific Industries, Bohemia, NY, USA) and centrifuged for phase separation. Supernatants were transferred into tubes with 0.4 mL of chloroform:isoamilic-alcohol 24:1 pH 8 and centrifuged for phase separation. Supernatants were incubated overnight at −20 °C with 1 volume of isopropanol and 0.1 volume of sodium acetate 3 M. Precipitated DNA was pelleted by centrifugation, washed with cold ethanol twice and dried at 37 °C for ethanol evaporation. DNA was resuspended with Nuclease-free water (IDT, Coralville, IA, USA) and quantified using a NanoQuant Plate in a Tecan Infinite microplate reader (Tecan Trading AG).

### 4.6. Quantification of Bacterial Abundance by qPCR

Extracted DNA was diluted to 10 ng·μL^−1^, and used in qPCR reactions using 0.2 μM of the following primers [60]: for *Ba. vulgatus*, *Bacteroidetes* primer F (5′-GGTGTCGGCTTAAGTGCCAT-3′) and *Bacteroidetes* primer R (5′-CGGACGTAAGGGCCGTGC-3′); for *L. acidophilus*, LACTO_F (5′-TGGAAACAGRTGCTAATACCG-3′) and LACTO_R (5′-GTCCATTGTGGAAGATTCCC-3′); for *Bi. infantis*, BINF_17219F (5′-AGGTTACTTCGACGCCTTCT-3′) and BINF_17219R (5′-AGGTATTCGGTGACCAGCTT-3′), targeting a carbohydrate ABC transporter substrate-binding protein; and for *E. coli*, 0.1 μM of the following primers: Eco1457F (5′-CATTGACGTTACCCGCAGAAGAAG) and Eco1652R (5′-CTCTACGAGACTCAAGCTTGC-3′). qPCR reactions were performed using the qPCR SensiFAST SYBR No-ROX kit (Bioline, London, UK) in MicroAmp Fast Optical plates (Applied Biosystems, Foster City, CA, USA), and using an AriaMx Realtime PCR System machine (Agilent Technologies). Reactions were carried out with an initial cycle for polymerase activation for 2 min at 50 °C and 2 min at 95 °C, followed by 40 cycles of 3 s at 95 °C, 10 s at 62 °C and 20 s at 72 °C. Absolute quantification was performed including a standard curve using DNA from a pure culture of each species, with dilutions starting from 10 ng·μL^−1^ to 0.1 pg·μL^−1^. To convert quantified bacterial DNA amounts into genome copy numbers, the following equation was used. Genome sizes and rRNA copy numbers are indicated in Table S2.

(2) $$Cell\;copies/mL=\frac{Avogadro\;N{}^{\circ}\;\left({mol}^{-1}\right)\times DNA\;quantity\left(g/mL\right)\times Genome\;16S\;copy\;number\;}{Genome\;size\;\left(pb\right)\times 660\left(\frac{g}{mol}\right)}\;$$

Cell copies for each microbe during the co-culture experiments were added and the percentage of each microbe in each co-culture was determined as:

(3) $$Percentage\;bacterium\;i=\frac{Cell\;copies\;bacterium\;i\;}{Cell\;copies\;bacterium\;i+Cell\;copies\;bacterium\;j}\times 100\;$$

Data represent the averages of two biological duplicates and three technical replicates.

### 4.7. Substrate Consumption

Total carbohydrate concentration in supernatants from all experiments was assayed using the microplate assay phenol-sulphuric acid method [61]. Briefly, 30 μL of a sample, previously diluted with distilled water to 1:20 for 2-FL and 1:125 for FOS, were loaded into the wells of a 96-well microplate (Corning Incorporated, Corning, NY, USA). 100 μL of sulfuric acid 98% and 20 μL of phenol 5% were added to the samples, and microplates were incubated for 5 min at 90 °C into a thermostatic water cabinet (Quimis, Diadema, Brazil). The microplate was cooled in ice-water for 5 min. Absorbance at 490 nm was measured using a Tecan Infinite microplate reader (Tecan Trading AG) and compared to a standard curve using the same substrate. Total consumption was calculated as percentage relative to time 0. Data was expressed as oligosaccharide consumption (100%—%relative carbohydrate concentration left).

Protein concentration in supernatant was estimated by the Bradford assay, using the Bio-Rad Protein Assay Dye (Bio-Rad, Hercules, CA, USA). 10 μL of sample were added and absorbance was measured at 595 nm in a Tecan Infinite microplate reader (Tecan Trading AG). Protein concentration was estimated using the observance value obtained, contrasted against a standard curve made with dilutions of Bovine Serum Albumin starting from 1 to 0.1 mg·mL^−1^. Protein concentrations were expressed as a percentage relative to the initial amounts.

### 4.8. SCFA Quantification

Acetate and lactate production in supernatants were measured by HPLC using a Lachrom L-700 HPLC system (Hitachi, Tokyo, Japan), equipped with a Diode Array and Refractive Index detectors (Hitachi), as previously described [62]. Supernatants corresponding to the same biological duplicate were pooled in one sample. Organic molecules were separated using an Aminex HPX-87H ion exchange carbohydrate-organic acid column (Bio-Rad), with a flux of 0.45 mL/min of H_2_SO_4_ 5 mM with an oven temperature of 35 °C. Samples were quantified using a standard curve made with dilutions of l-(+)-Lactic acid (Sigma) and sodium acetate (Sigma) starting from 30 to 0.07 g·L^−1^.

## 5. Conclusions

In conclusion, in this study we have designed a culture medium that, resembling better infant gut conditions, was used to study microbial interactions in a consortium of the infant gut microbiome. These interactions were mostly competitive, and affected bacterial abundance, prebiotic consumption and SCFA production. Obtaining different microbial interactions within the same consortium of species using either 2FL or FOS suggests that the chemical nature of a prebiotic influences which microbial interactions will be predominant. Moreover, the interactions observed are complex and are not necessarily representative of bacterial single growth.

## Supplementary Materials

Supplementary materials can be found at www.mdpi.com/1422-0067/18/10/2095/s1.
