# Supplementary material for: Prebiotics Mediate Microbial Interactions in a Consortium of the Infant Gut Microbiome
Source: Int J Mol Sci. 2017 Oct 4;18(10):2095. doi: 10.3390/ijms18102095 (PMC5666777; doi:10.3390/ijms18102095)
Supplement: Supplementary file 1 [file ijms-18-02095-s001.pdf]

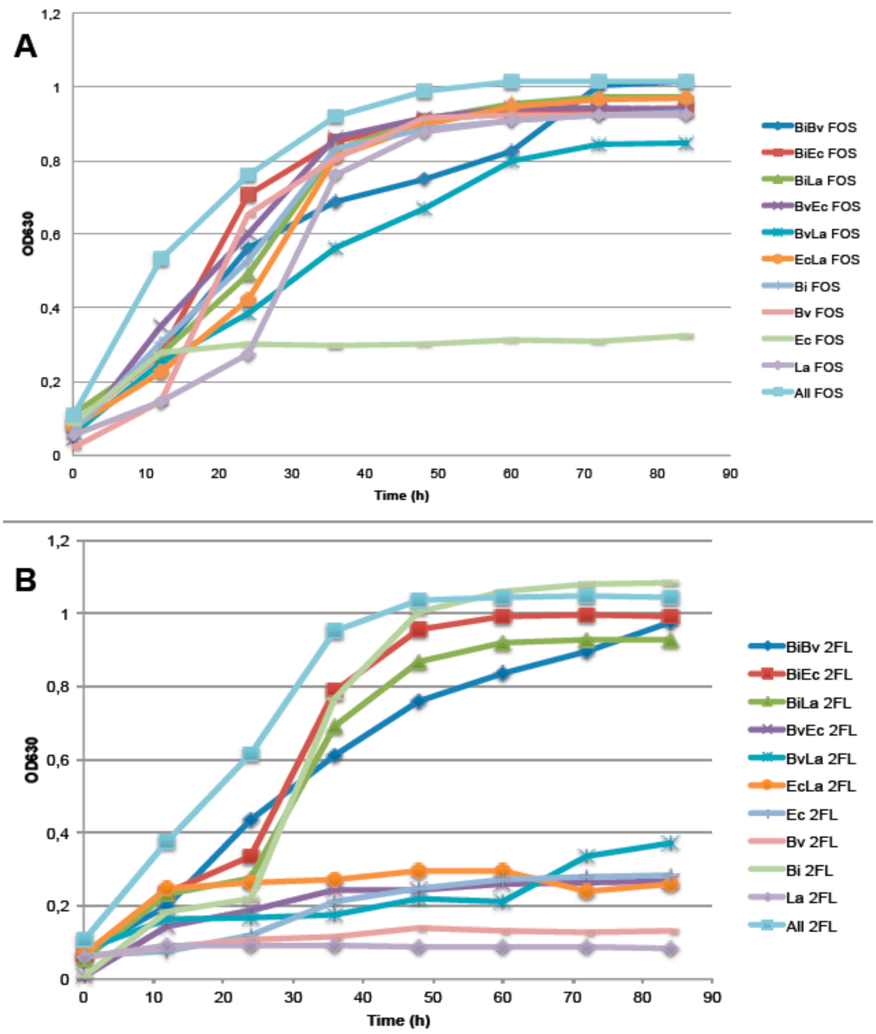


**Figure S1.** Bacterial growth of mono and co-cultures. Combinations of *Bi. infantis* (Bi), *Bv. vulgatus* (Bv), *L. acidophilus* (La) and *E. coli* (Ec) were grown in either FOS (A) or 2FL(B).

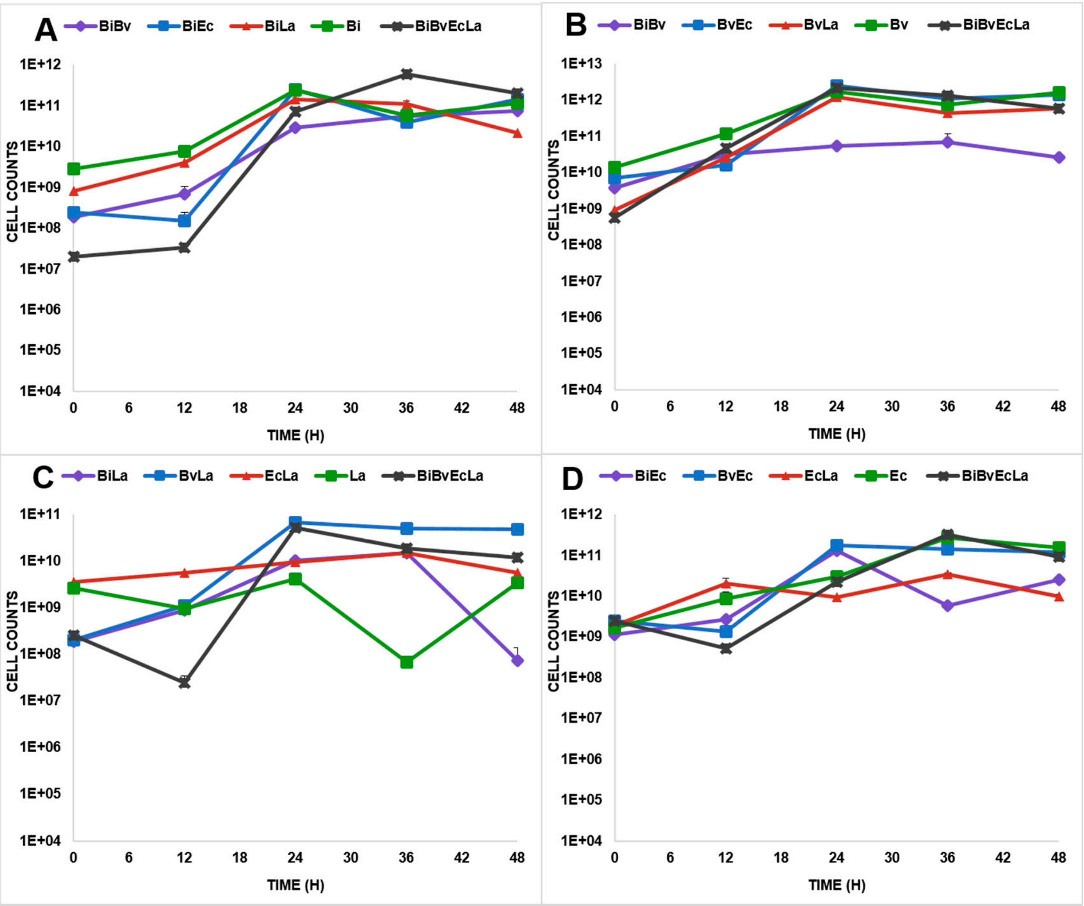


**Figure S2.** Bacterial abundance in co-culture during growth on 2FL. Numbers indicate cell counts for each bacterium in mono or co-culture. A: *Bi. infantis* combinations; B: *Bv. vulgatus* combinations; C: *L. acidophilus*; D: *E. coli* combinations. Error bars indicate the standard error of the media.

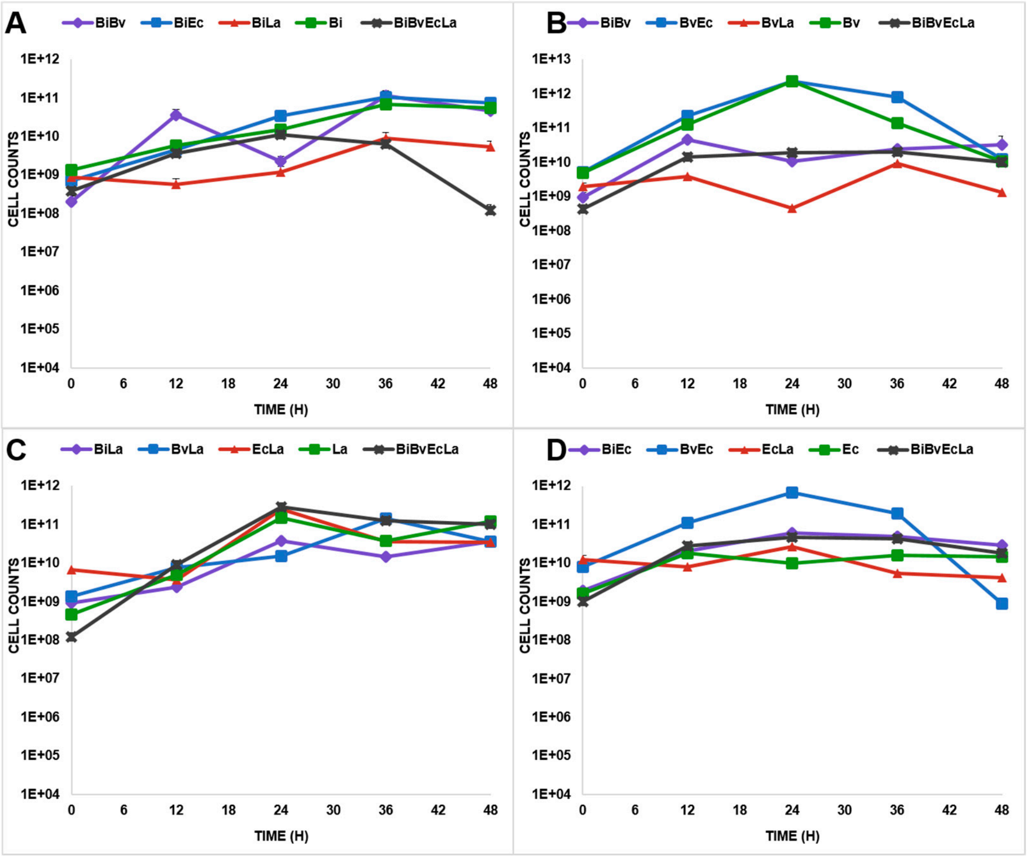


**Figure S3.** Bacterial abundance in co-culture during growth on FOS. Numbers indicate cell counts for each bacterium in mono or co-culture. A: *Bi. infantis* combinations; B: *Bv. vulgatus* combinations; C: *L. acidophilus*; D: *E. coli* combinations. Error bars indicate the standard error of the media.

## SUPPORTING INFORMATION

## Table S1: Final composition of mZMB.

| Description | Group Nº | Component | Final Concentration [g/L] |
|---|---|---|---|
| Vitamin | 8 | Myo-inositol | 0.002 |
| Phosphate Buffer | 9 | $KH_2PO_4$ | 3.144 |
| | | $K_2HPO_4$ | 6.426 |
| Important Vitamin Group | 11 | Calcium pantothenate | 0.0012 |
| | | Niacin | 0.0009 |
| | | Pyridoxal HCL | 0.0048 |
| Important Mineral Group | 12 | $MgSO_4*7H_2O$ | 1 |
| | | $FeSO_4*7H_2O$ | 0.004 |
| | | $ZnSO_4*7H_2O$ | 0.005 |
| Other Vitamin Group | 13 | Folic Acid | 0.0005595 |
| | | p-Aminobenzoic acid | 0.00005595 |
| Fatty acid Group | 14 | Potassium acetate | 0.901 |
| | | Thioctic acid | 0.001 |
| Nucleic Acid Base Group | 15 | Adenine | 0.01101 |
| | | Guanine | 0.005505 |
| | | Uracil | 0.02202 |
| | | Xanthine | 0.00367 |
| Trace Mineral Group | 17 | $(NH_4)_6Mo_7O_2*4H_2O$ | 0.00019 |
| | | $MnSO_4*4H_2O$ | 0.00038 |
| | | $CaSO_4*4H_2O$ | 0.038 |
| | | $CoSO_4*6H_2O$ | 0.000228 |
| | | $CuSO_4*5H_2O$ | 0.00019 |
| | | $H_3BO_3$ | 0.00076 |
| | | $K_2SO_4$ | 0.0228 |
| | | KI | 0.000114 |
| Chelator Group | 18 | EDTA | 0.0075 |
| | | Nitriliotriacetic Acid | 0.0075 |
| Other Component Group | 19 | Glutathione | 0.015 |
| | | $(NH_4)_2SO_4$ | 1 |
| | | NaCl | 3 |
| EAA#8 | 21 | Biotin | 0.006 |
| EAA#9 | 22 | Thiamine | 0.0005595 |
| EAA#10 | 23 | Riboflavin | 0.0009 |
| Tryptone | | 32.4 | |
| 2-Fucosyllactose/FOS | | 10 | |
| Bile Salts | | 0.05 | |
| Cysteine-HCl | | 1 | |
| Mucin | | 4 | |
| Hemin | | 0.01 | |

**Table S2. Genome sizes and 16S rRNA copy numbers for the four bacteria used in this study.**

| Species | Genome size (pb) | 16S rRNA Copy Nº |
|---|---|---|
| *Bacteroides vulgatus* ATCC8482 | 5.39E+06 | 7 |
| *Bifidobacterium infantis* ATCC15697 | 2.83E+06 | 4 |
| *Escherichia coli K12* | 4.65E+06 | 7 |
| *Lactobacillus acidophilus* UCD326 | 2.00E+06 | 1 |

**Table S3.** Ratio of growth rates for bacteria in the first column in co-culture with microorganisms in the first row, to the growth rate in monoculture. This was calculated as log(Xi/Xo)/12, corresponding to the change in biomass between time 12 and 24. Growth rate in monoculture is also indicated.

| **2FL** | **Bi** | **Bv** | **Ec** | **La** | **All** | **umax** |
|---|---|---|---|---|---|---|
| **Bi** | 1.00 | 1.08 | 2.15 | 1.04 | 2.21 | 0.22 |
| **Bv** | 0.20 | 1.00 | 1.89 | 1.43 | 1.45 | 0.30 |
| **Ec** | 3.04 | 3.83 | 1.00 | -0.64 | 2.94 | 0.11 |
| **La** | 1.66 | 2.82 | 0.36 | 1.00 | 5.25 | 0.12 |
| | | | | | | |
| **FOS** | **Bi** | **Bv** | **Ec** | **La** | **All** | **umax** |
| **Bi** | 1.00 | -0.03 | 1.04 | 1.11 | 0.35 | 0.24 |
| **Bv** | -0.51 | 1.00 | 0.80 | -0.73 | 0.09 | 0.19 |
| **Ec** | 0.92 | 1.54 | 1.00 | 0.49 | 0.21 | 0.20 |
| **La** | 0.80 | 0.20 | 1.24 | 1.00 | 1.01 | 0.28 |

**Table S4. Protein consumption during growth on 2FL or FOS.**

| **2FL** | **0** | **SD** | **12** | **SD** | **24** | **SD** | **36** | **SD** | **48** | **SD** |
|---|---|---|---|---|---|---|---|---|---|---|
| **BiBv** | 100.00 | 4.21 | 106.80 | 19.13 | 81.52 | 19.78 | 70.83 | 16.39 | 78.59 | 3.98 |
| **BiEc** | 100.00 | 2.30 | 80.76 | 35.36 | 86.72 | 13.04 | 86.06 | 3.47 | 92.94 | 3.58 |
| **BiLa** | 100.00 | 2.44 | 82.85 | 3.73 | 96.69 | 5.81 | 86.36 | 53.46 | 97.99 | 11.42 |
| **BvEc** | 100.00 | 14.31 | 122.98 | 5.19 | 94.43 | 0.31 | 78.31 | 0.89 | 71.28 | 22.68 |
| **BvLa** | 100.00 | 3.91 | 93.91 | 4.50 | 96.84 | 13.76 | 80.49 | 12.98 | 77.57 | 27.59 |
| **EcLa** | 100.00 | 19.65 | 92.70 | 8.06 | 94.66 | 14.25 | 76.46 | 4.31 | 64.56 | 18.41 |
| **Bi** | 100.00 | 8.03 | 110.26 | 11.91 | 92.11 | 3.31 | 115.24 | 13.12 | 119.63 | 18.69 |
| **Bv** | 100.00 | 1.20 | 96.34 | 6.62 | 97.45 | 23.42 | 89.23 | 18.75 | 64.01 | 8.05 |
| **Ec** | 100.00 | 1.82 | 100.00 | 0.16 | 114.85 | 2.90 | 108.31 | 13.11 | 68.31 | 2.48 |
| **La** | 100.00 | 1.96 | 111.98 | 10.42 | 106.18 | 5.53 | 125.49 | 47.50 | 78.36 | 13.45 |
| **BiBvEcLa** | 100.00 | 0.08 | 105.80 | 13.77 | 93.98 | 4.73 | 76.33 | 19.15 | 96.48 | 39.59 |
| | | | | | | | | | | |
| **FOS** | **0** | **SD** | **12** | **SD** | **24** | **SD** | **36** | **SD** | **48** | **SD** |
| **BiBv** | 100.00 | 3.95 | 107.73 | 0.34 | 96.56 | 1.52 | 97.55 | 0.09 | 98.34 | 4.17 |
| **BiEc** | 100.00 | 3.19 | 86.09 | 7.00 | 126.07 | 2.39 | 100.94 | 7.26 | 110.14 | 2.17 |
| **BiLa** | 100.00 | 3.40 | 88.22 | 7.72 | 78.53 | 3.87 | 82.80 | 20.97 | 86.79 | 0.69 |
| **BvEc** | 100.00 | 1.71 | 101.59 | 5.30 | 104.06 | 7.16 | 109.90 | 3.49 | 113.09 | 1.33 |
| **BvLa** | 100.00 | 2.35 | 96.87 | 9.80 | 96.87 | 62.92 | 92.66 | 2.81 | 92.90 | 7.33 |
| **EcLa** | 100.00 | 1.89 | 95.51 | 0.36 | 102.91 | 5.50 | 88.39 | 0.63 | 86.02 | 8.62 |
| **Bi** | 100.00 | 4.96 | 89.42 | 9.83 | 94.28 | 0.18 | 91.47 | 7.69 | 93.13 | 9.21 |
| **Bv** | 100.00 | 6.41 | 99.62 | 2.08 | 106.70 | 6.60 | 103.64 | 4.94 | 102.99 | 3.10 |
| **Ec** | 100.00 | 0.72 | 105.12 | 2.75 | 117.77 | 7.22 | 100.27 | 0.75 | 104.25 | 0.48 |
| **La** | 100.00 | 0.45 | 103.84 | 0.79 | 86.80 | 1.36 | 93.26 | 1.44 | 108.38 | 10.26 |
| **BiBvEcLa** | 100.00 | 2.83 | 107.18 | 3.37 | 120.43 | 13.05 | 113.17 | 10.26 | 112.27 | 7.43 |
